# Genetic diversity and phylogeny of avian orthoreovirus isolates from Peruvian broiler chickens

**DOI:** 10.1128/mra.00054-25

**Published:** 2025-04-04

**Authors:** Doris Villanueva-Pérez, Luis Tataje-Lavanda, Angela Montalván-Avalos, Gisela Isasi-Rivas, Diego Paredes-Inofuente, Suly Montoya-Ortiz, Manolo Fernández-Sánchez, Eliana Icochea, Manolo Fernández-Díaz

**Affiliations:** 1 Laboratorios de Investigación y Desarrollo, FARVET, Chincha Alta, Ica, Peru; 2 Escuela Profesional de Medicina Humana, Universidad Privada San Juan Bautista 33222, Lima, Peru; 3 Laboratorio de Patología Aviar, Facultad de Medicina Veterinaria, Universidad Nacional Mayor de San Marcos 33209, Lima, Peru; DOE Joint Genome Institute, Berkeley, California, USA

**Keywords:** Avian orthoreovirus, viral arthritis, σC gene, phylogenetic, Peru, poultry, Nanopore Sequencing

## Abstract

Avian orthoreovirus infections are increasingly common in Peruvian broiler chickens, primarily causing arthritis/tenosynovitis syndrome. In this study, sequencing and phylogenetic analysis of the σC gene revealed that avian orthoreovirus isolates were clustered in genotypes 1, 2, and 3. However, further research is needed to understand its genetic diversity and improve vaccination strategies.

## ANNOUNCEMENT

Avian orthoreovirus or avian reovirus (ARV) causes viral arthritis/tenosynovitis in commercial poultry, affecting productivity. ARV belongs to the *Orthoreovirus* genus, subfamily *Spinareovirinae* of the *Reoviridae* family and has a genome of 10 double-stranded RNA segments (1–3). The S1 segment encodes the σC gene, crucial for viral attachment, immune response (3, 4), and genotypic classification (2, 5, 6). To date, ARV genotypes in Peru remain unidentified.

This study sequenced and analyzed the σC gene of ARV isolates from Peruvian broilers. Eight tendon samples were collected in March 2024 from broilers with viral arthritis symptoms in poultry-producing regions of Peru (Table 1). ARV detection was conducted using real-time RT-PCR targeting the S3 gen (7). The PCR-positive samples were minced, homogenized, and centrifuged. The supernatant was filtered and used to infect LMH cells. After 5 days, cells showing >90% cytopathic effects were collected and clarified, and supernatants were conserved at −80°C (4). RNA was extracted from 140 µL supernatant using the QIAamp Viral RNA Mini Kit (Qiagen, Germany), followed by reverse transcription with ProtoScript II Reverse Transcriptase (New England Biolabs, USA) and random hexamers according to the manufacturer’s protocol. Subsequently, the σC gene was amplified using primers P1 and P4 (2) and Q5 High-Fidelity 2× Master Mix (New England Biolabs, USA). PCR products (_~_1,088 bp) were purified using the QIAquick Gel Extraction Kit (Qiagen, Germany), quantified, and prepared for sequencing using the ONT Rapid Barcoding Kit 24 (SQK-RBK114.24).

**TABLE 1:** Genotypic and BLASTn analysis of ARV isolates obtained from broiler chickens

Isolate name (accession number/SRA)	Age of host (days)	Origin (region)	Genotypic cluster	Reference sequence	Consensus length (pb)	σC gene length/% GC	BLASTn coverage/ identity (%)	Closest isolate (accession number/country)
VFAR-196 (PQ660261/SRX27158824)	28	Arequipa	1	ON782394.1	1101	981/48	100/99.55	Reo/ZC0219/SD/2017 (PQ336715.1/China)
VFAR-197 (PQ660262/SRX27158825)	35	La Libertad	1	AF330703.1	1101	981/48	100/99.46	Reo/ZC0219/SD/2017 (PQ336715.1/China)
VFAR-159 (PQ660254/SRX27158818)	8	La Libertad	2	MT161583.1	963	963/48.1	99/94.9	USP_BR_362-5 (MT161583.1/Brazil)
VFAR-160 (PQ660255/SRX27158819)	14	La Libertad	2	MT161583.1	963	963/48.1	99/95.94	USP_BR_362-5 (MT161583.1/Brazil)
VFAR-161 (PQ660256/SRX27158820)	14	La Libertad	2	MN879660.1	1089	981/47.4	99/84.23	USP_BR_362-5 (MT161583.1/Brazil)
VFAR-162 (PQ660257/SRX27158821)	10	Arequipa	2	MT161583.1	963	963/48.1	99/94.9	USP_BR_362-5 (MT161583.1/Brazil)
VFAR-193 (PQ660259/SRX27158818)	21	Arequipa	2	MT161583.1	963	963/48.2	99/95.73	USP_BR_362-5 (MT161583.1/Brazil)
VFAR-175 (PQ660258/SRX27158822)	21	Arequipa	3	OP816520.1	768	768/46	100/92.71	D7 (OP816520.1/Canada)

Sequencing was performed on a MinION Mk1B device (Oxford Nanopore Technologies, UK) using an R10.1 flow cell (N50: 858). Basecalling and demultiplexing were conducted using Dorado Software v7.4.12 (8) with Fast model v4.3.0, 400 bps, generating 270.21 k reads and 155.9 Mb of high-quality bases. Initially, processed reads were aligned to reference sequences representing diverse ARV genotypes using the GalaxyTrakr platform (https://galaxytrakr.org/) (9); all tools were run with default parameters unless otherwise specified. Quality control was assessed using tools such as FastQC (10) (Galaxy Version 0.73+galaxy0), Porechop (11) (Galaxy Version 0.2.4+galaxy0), NanoFilt (12) (Galaxy Version 0.1.0), and MultiQC (13) (Galaxy Version 1.11+galaxy1). Consensus sequences were generated from each alignment and subsequently compared against ARV references (GenBank: AF330703.1, MT161583.1, MN879660.1, ON782394.1, OP816520.1) (Table 1) using Map with BWA-MEM (14) (Galaxy Version 0.7.17.2), Samtools depth (15) (Galaxy Version 1.15.1+galaxy0), and Consensus Sequence (16) (Galaxy Version 0.7.0+galaxy1). The closest matching isolate sequences were identified via BLASTn (17, 18) (Table 1). This approach improved consensus sequence accuracy by minimizing SNPs, indels, and frameshift errors, ensuring a more precise genotype representation. Phylogenetic analysis was performed on eight σC gene sequences using MEGA 11 v 11.0.13 (19). Multiple sequence alignments of nucleotides were generated with MUSCLE (20), and a neighbor-joining phylogenetic tree was constructed with 1,000 bootstrap replicates. Evolutionary distances were computed using the Tajima-Nei method with gamma distribution modeling (shape parameter = 1). The analysis involved 82 nucleotide sequences and 499 aligned positions in the final data set.

**Fig 1:**
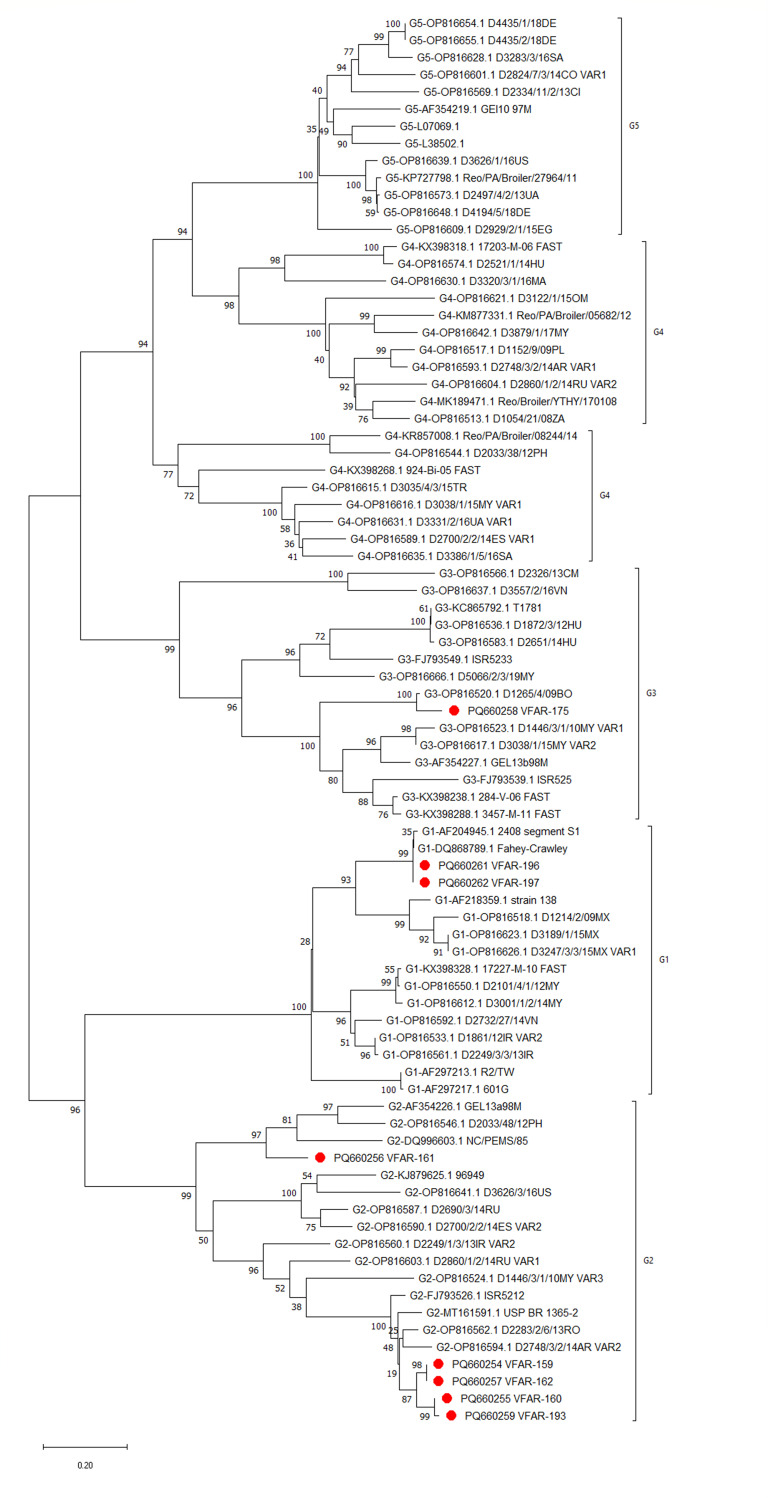
Phylogenetic tree of the σC gene sequences of avian orthoreovirus isolates from Peruvian broiler chickens.

The results showed consensus sequence lengths of these σC genes range from 768 bp to 981 bp (Table 1). Phylogenetic analysis revealed three distinct genotypes (1, 2, and 3) circulating among Peruvian broilers (Fig. 1). Genotype 2, closely related to Brazilian isolates, was the most prevalent among ARV isolates, suggesting epidemiological links. The diversity highlights the need for continued surveillance to guide vaccine strategies.

Phylogenetic analysis revealed five distinct clades, with the eight Peruvian ARV isolates clustering into genotypes 1, 2, and 3. Isolates were highlighted with red circles for visual distinction. The tree was constructed using the neighbor-joining method with 1,000 bootstrap replicates, and branch lengths represent evolutionary distances calculated using the Tajima-Nei method. A gamma distribution (shape parameter = 1) was applied to model rate variation among sites. The data set included 82 sequences, with 499 positions after gap and missing data elimination. Bootstrap values are indicated at branch nodes.

## Data Availability

The samples were uploaded to the National Center for Biotechnology Information under BioProject accession number PRJNA1201241. The accession numbers for all eight SRA files are as follows: SRX27158818–SRX27158825.
